# Targeting LC3B/MCL-1 expression by Protodioscin induces autophagy and apoptosis in human glioblastoma cells *in vitro* and *in vivo*

**DOI:** 10.7150/ijms.116656

**Published:** 2025-08-22

**Authors:** Ching-Ting Tai, Yi-Hsien Hsieh, Hsiang-Lin Lee, Pei-Ni Chen, Hui-Ling Chiou, Tsai-Yun Wu, Chia-Liang Lin, Yi-Chen Lin, Chien-Min Chen

**Affiliations:** 1 Institute of Medicine, Chung Shan Medical University, Taichung, Taiwan.; 2 Department of Medical Research, Chung Shan Medical University Hospital, Taichung, Taiwan.; 3 School of Medicine, Chung Shan Medical University, Taichung, Taiwan.; 4 Deptartment of Surgery, Chung Shan Medical University Hospital, Taichung, Taiwan.; 5 School of Medical Laboratory and Biotechnology, Chung Shan Medical University, Taichung, Taiwan.; 6 Department of Biochemistry, School of Medicine, Chung Shan Medical University, Taichung, Taiwan.; 7 Clinical Laboratory, Chung Shan Medical University Hospital, Taichung, Taiwan.; 8 Division of Neurosurgery, Department of Surgery, Changhua Christian Hospital, Changhua, Taiwan.; 9 Department of Biomedical Sciences, National Chung Cheng University, Chiayi, Taiwan.

**Keywords:** Protodioscin, Autophagy, Apoptosis, Glioblastoma, LC3, MCL-1

## Abstract

Protodioscin (PD), a natural steroidal saponin extracted from Dioscorea and Tribulus terrestris, has been shown to exhibit anticancer, antimetastatic, and pro-autophagic and apoptotic activities in various malignant tumor cells. However, its antitumor potential and molecular mechanisms in glioblastoma remain unclear. This study aimed to investigate the effects of PD on apoptosis and autophagy in human glioblastoma cells using both *in vitro* and *in vivo* models. Our results suggested that PD significantly inhibited the proliferation, induced mitochondrial dysfunction and apoptosis of human GBM8401 and M059K cells, as evidenced by the activation of cleaved-PARP (c-PARP) and MCL-1 expression. However, PD also enhanced autophagic activity, as indicated by the upregulation of LC3B expression. Silencing LC3 expression using siRNA markedly attenuated PD-induced autophagy and apoptosis, these results demonstrated the crucial regulatory role of LC3 in mediating cell death pathways. In an *in vivo* GBM8401 xenograft model, PD treatment significantly suppressed GBM8401 tumor growth without affecting body weight or causing organ toxicity in PD-treated mice. Immunohistochemical analysis further suggested that PD reduced the expression of the Ki67 expression in glioblastoma tumor tissues and molecular docking finding that PD strong interaction with LC3B and MCL-1. In summary, PD induces both apoptosis and autophagy in glioblastoma cells through LC3B/MCL-1 modulation, demonstrating strong antitumor potential against human glioblastoma. These findings suggest that PD may serve as a novel therapeutic strategy and a promising candidate for glioblastoma treatment.

## Introduction

Human glioblastoma is one of the most dangerous and difficult human tumors to treat due to its rapid growth rate and resistance to chemotherapeutic drugs. The median survival time for most patients is 15 months because of the lack of effective clinical treatment 1. Current treatment mainly consists of conventional chemotherapy drugs that inhibit glioblastoma cell proliferation. Recently, the use of biological agents for glioblastoma treatment has gained widespread attention in clinical practice. Many natural compounds are reported to possess anti-tumor activity and target relevant pathways to exert strong anti-cancer effects 2. However, the effectiveness of current glioblastoma treatments remains limited, with more than 50% of patients opting for complementary and alternative therapies, of which natural medicine is one of the most commonly used approaches 3.

PD is a natural steroidal saponin with broad biological activity, extracted from the *Tribulus terrestris* and *Dioscorea spp* 4. PD exhibits a variety of biological activities, including anti-tumor, antioxidant, and cell death effects 5, 6. Effects on specific cancer cells include the induction of mitochondrial autophagy in osteosarcoma cells 7, increasing oxidative stress to inhibit breast cancer cell growth 8, and promoting endoplasmic reticulum stress-induced mitochondrial apoptosis in hepatocellular carcinoma cells 9. However, the functional role and underlying mechanisms of PD action in glioblastoma cells remain unclear.

During the autophagy process, cytosolic LC3-A conjugates with phosphatidylethanolamine (PE) to form the LC-3B conjugate, which is then incorporated into autophagosomes. Subsequently, autophagosomes fuse with lysosomes to form autolysosomes, leading to the degradation of LC3-II within the autolysosomal lumen 10. Thus, LC3 serves as an important protein for monitoring cellular autophagy and autophagic cell death 11. Previous studies have demonstrated that LC3B plays a crucial biological role in a variety of physiological and pathological processes and is associated with tumorigenesis, immune diseases, cardiovascular diseases, and inflammatory responses 12. Furthermore, LC3B play an important role in regulatory mechanisms involved in tumor formation, metastasis, and chemotherapy resistance 13. Additionally, the action of several natural compounds that induce cell death is closely associated with LC3B expression in tumor cells, including Hinokitiol-induced apoptosis and autophagy of breast cancer stem cells through the CD44/Nanog/SOX2/Oct4 pathway 14. One study has shown that PD induces mitophagy through the NIX/LC3 pathway, leading to apoptosis in human osteosarcoma cells 7. However, the effects of PD on human glioblastoma cells remains unclear.

In this study, we investigated the anti-tumor effects of PD on three glioblastoma cell lines, evaluating its ability to inhibit glioblastoma cell growth and the underlying molecular mechanisms. In addition, a glioblastoma mouse model was used to assess the *in vivo* antitumor efficacy of PD and evaluate its drug safety.

## Materials and Methods

### Chemicals, drugs, and reagents

PD (C_51_H_84_O_22_; MW, 1049.21 g/mol) was purchase from Chemface company (Hubei, PRC). The annexin V & Dead Cell Kit (MCH100105) and MitoPotential Kit (MCH100110) were purchased from Luminex Corporation (Austin, TX 78727, USA). MTT ((3-(4,5-Dimethylthiazol-2-yl)-2,5-diphenyltetrazolium bromide) and acridine orange reagent (AO) were purchased from Sigma-Aldrich (Louis, MO, USA). Lipofectamine RNAiMAX transfection reagent was purchased from Thermo Fisher Scientific Inc (Waltham, MA, USA). The LC3B antibodies (NB100-2220; 1:1000) were purchased from Novus Biologicals (Centennial, CO, USA). The Ki-67 (ab15580; 1:100) antibody used for IHC staining was purchased from Abcam plc (Cambridge, UK). For western blot analysis, we purchased active-form PARP (c-PARP; #9542, 1:1000) and MCL-1 (#94296; 1:1000) antibodies from Cell Signaling Technology Inc. (Danvers, MA, USA), and GAPDH (60004-1-Ig; 1:5000) antibodies from Proteintech Group, Inc (Rosemont, IL, USA). Pan-caspase inhibitor (Z-VAD) was purchased from Santa Cruz Biotechnology (Santa Cruz, CA, USA).

### Cell culture

GBM8401 (glioblastoma multiforme; BCRC 60163) was cultured in RPMI 1640 medium. M059K (glioblastoma; BCRC 60381) was cultured in DMED/F12 (Dulbecco's Modified Eagle's and Ham's F12) medium, supplemented with sodium pyruvate (0.5 mM) and non-essential amino acids (NEAA, 0.05 mM). U251 cells (glioblastoma) were maintained in MEM (Eagle's minimal essential) medium supplemented with fetal bovine serum and penicillin-streptomycin solution.

### Growth of human glioblastoma cells

The three glioblastoma cell lines (2 × 10^4^ cells) were treated with PD (4-24 μM) for 24 hours, followed by the addition of MTT reagent (1 μg/mL) for 4 hours. Formazan was dissolved in 100% DMSO, and the absorbance (OD 595 nM) was determined using an ELISA reader 15.

### Annexin V/PI assay of cell apoptosis

Human glioblastoma cells were treated with PD (0.5, 1, and 2 μM) for 24 hours. Cells then were washed with ice-cold PBS twice and treated with the Annexin V & Dead Cell reagents for 15 minutes in the dark. After washing twice with PBS, the fraction of apoptotic cells (%) was determined using a Guava Muse flow cytometer (Cytek, Fremont, CA, USA).

### Mitochondrial membrane potential

Human glioblastoma cells were treated with PD (0.5, 1, and 2 μM) for 24 hours. The MitoPotential Kit was used to treat the cells for 15 minutes in the dark. Cells with loss of mitochondrial membrane potential were detected using the Guava Muse flow cytometer (Cytek, Fremont, CA, USA).

### Assessment of autophagy by acridine orange staining

Human glioblastoma cells (2×10⁵ cells/well) were seeded into 6-well plates, incubated overnight at 37°C, and treated with PD (6, 12, and 18 µM) for 24 hours. The culture medium then was discarded, and the cells were washed for 5 minutes three times with phosphate-buffered saline (PBS). Subsequently, cells were incubated with acridine orange staining solution (final concentration, 5 μg/mL) at 37°C in the dark for 15 minutes and then rinsed three times with PBS. Coverslips were gently removed, mounted onto glass slides, and counterstained with DAPI. Acidic vesicular organelles (AVOs) were then visualized using a fluorescence microscope (emission Wavelength at 650 nm) to evaluate autophagic activity.

### Small interfering RNA (siRNA) transfection assay

Human glioblastoma cells (2×10⁵ cells/well) were plated in 6-cm culture dishes and allowed to adhere overnight. siRNA targeting LC3B (si-LC3B; 20 nM) and control siRNA (si-Con; 20 nM) were each incubated in Opti-MEM for 5 mins. Lipofectamine RNAiMAX (for siRNA delivery) was incubated in Opti-MEM for 5 mins, followed by a 15-minute incubation after mixing to allow complex formation. The siRNA/Lipofectamine RNAiMAX-complex was then added to PD (18 µM)-treated glioblastoma cells in culture medium with 10% FBS, followed by incubation under standard culture conditions for an additional 24 hours before assays for cell growth or apoptosis.

### Clinical data analysis

The mRNA expression level of MCL1 in human glioblastoma tissues was compared with that in adjacent normal tissues using the Gene_DE module of the TIMER2.0 database (http://timer.cistrome.org/), based on clinical data from The Cancer Genome Atlas (TCGA).

### Western blot analysis

Total protein extracted from PD-treated glioblastoma cells in NETN buffer was placed on ice for 20 minutes. A total of 20 μg of protein (as determined by Bradford protein assay) was separated via 10% SDS-PAGE and then transferred to a PVDF membrane using a semi-dry transfer system for 1 hour. Membranes were blocked in TBST containing 2% BSA for 1 hour and then incubated overnight at 4°C with specific primary antibodies. Membranes then were washed with TBST buffer twice and then incubated for 1 hour with HRP-conjugated secondary rabbit or mouse antibodies. Chemiluminescent signals were developed using an ECL detection reagent and visualized using the ImageQuant LAS 4000 Mini imaging system (Cytiva, MA, USA). Band intensities were quantified by densitometry and normalized to GAPDH.

### *In vivo* tumorigenesis and immunohistochemistry assays

To evaluate the anti-tumorigenic potential of PD, a subcutaneous glioblastoma xenograft model was established using BALB/c nude mice (5-week-old females). GBM8401 cells (1 × 10⁶ cells in 100 μL of PBS mixed with Matrigel at a 1:1 ratio) were injected subcutaneously with a 24-gauge needle into the right flank of nude mice (n = 5). After 7 days and an increase in tumor to 85 mm^3^, tumor-bearing mice were orally administered PD (5 and 10 mg/kg) twice weekly. Tumor growth was assessed at 7-day intervals. After 5 weeks, the mice were euthanized and the tumors were excised, weighed, and photographed. Tumor tissues were then fixed in 10% neutral-buffered formalin for histological analysis by hematoxylin and eosin (HE) staining. Immunohistochemical assays were performed as previously reported 16. The Ki-67 antibody was used to assess cell proliferation. All animal experiments were conducted in accordance with the guidelines approved by the Institutional Animal Care and Use Committee (IACUC) of Chung Shan Medical University (Approval No. 2754).

### Statistical analysis

All experiments were repeated independently at least three times, and the data are presented as the mean ± standard deviation (SD). Statistical analyses were performed using GraphPad Prism software (version 6, GraphPad Software, Statistically Significant Inc., CA, USA). Results were compared between groups using one-way analysis of variance (ANOVA). Results were compared between two groups using Student's t-test. Statistical significance was considered as p < 0.05 or 0.01.

## Results

### PD inhibited the growth of human glioblastoma cells

**Figure 1:**
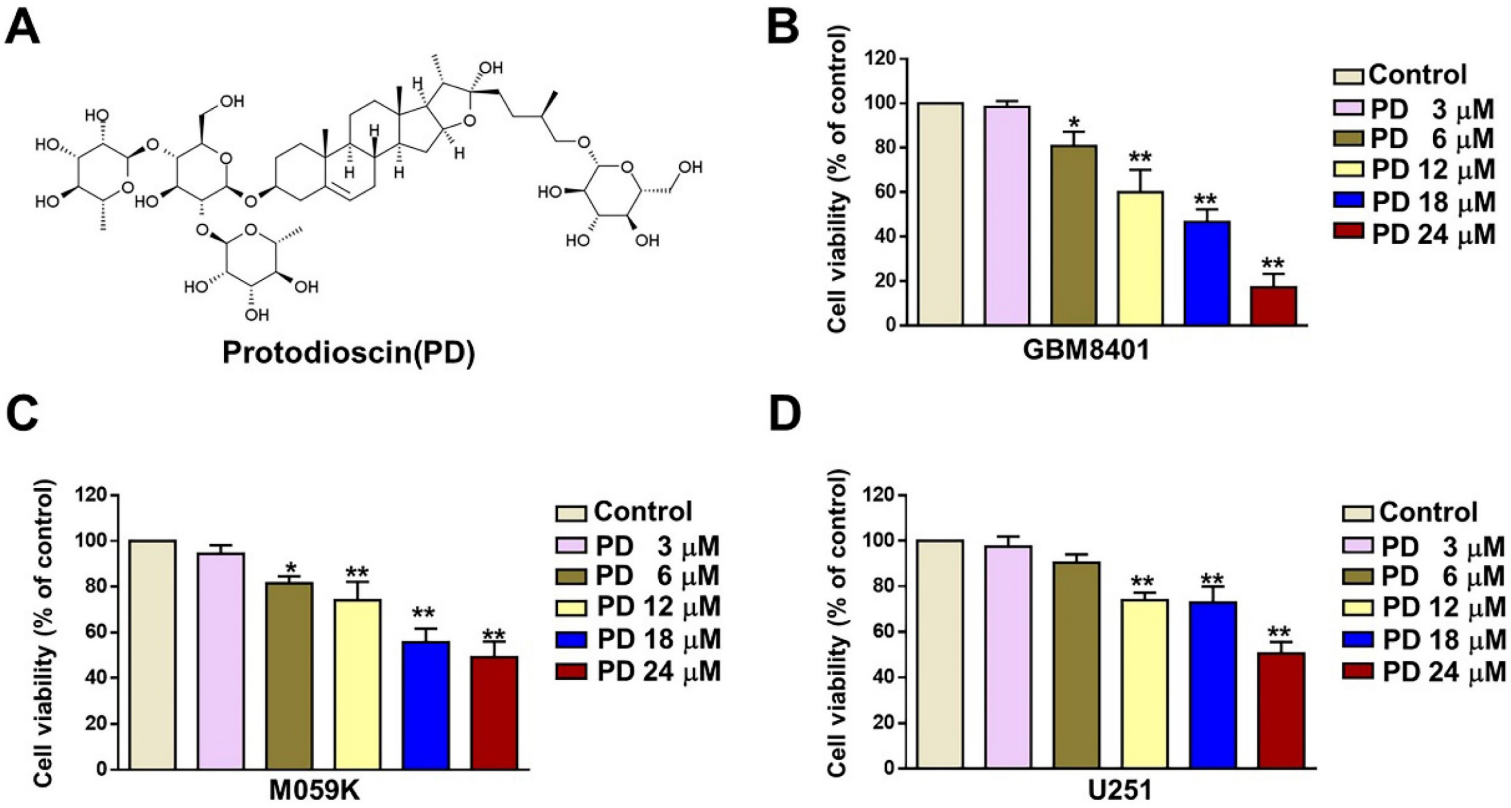
** Effect of PD on human glioblastoma cell growth.** (A) Chemical structure of Protodioscin (PD). (B-D) Three glioblastoma cell lines (GBM8401, M059K, and U-251) were treated with varying concentrations of PD for 24 hours. Cell growth was evaluated by MTT assay. Data are presented as the mean ± SD. *p < 0.05; ** p < 0.01 vs. control.

The structure of PD is shown in Figure 1A. To evaluate the anti-tumor effect of PD in human glioblastoma cells, we treated cells with various concentrations of PD (3, 6, 12, 18, and 24 μM) for 24 hours, and cell viability was assessed using the MTT assay. The results showed a significant reduction in GBM8401 and M059K cell growth (%) at concentrations of 6-24 μM PD (Figures 1B and 1C) and at 12-24 μM in U251 cells (Figure 1D). The IC_50_ values at 24 hours were 16.8 µM for the GBM8401 cells, the IC_50_ values were 20.8 µM for the M059K cells and IC_50_ value were 24.5 µM for the U251 cells, respectively. These findings indicate that PD effectively inhibits glioblastoma cell proliferation, and the optimal concentrations for further experiments were determined to be 6, 12, and 18 μM in all three glioblastoma cell lines.

### PD induced apoptosis in glioblastoma cells

**Figure 2:**
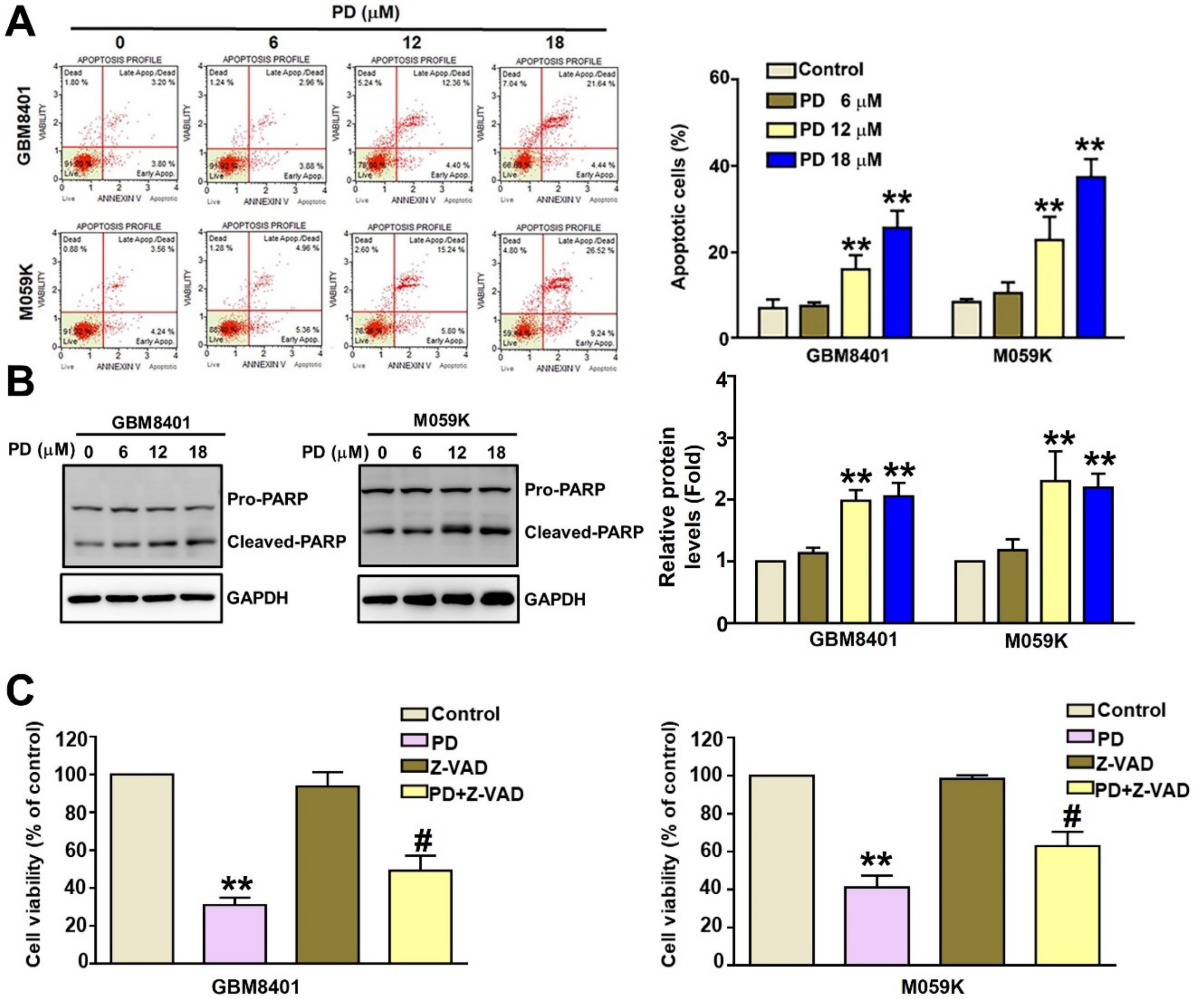
** PD induces apoptosis in human glioblastoma cells.** GBM8401 and M059K cells were treated with various concentrations of PD for 24 hours. (A) AnnexinV/PI staining assay was used to identify apoptotic cells. Right panel: quantification of apoptotic cells. (B) Western blot analysis of cleaved-PARP (c-PARP) expression. Right panel: quantification of fold-changes in c-PARP/GAPDH. (C) Cells were treated with PD, Z-VAD (20 µM), or PD +Z-VAD (20 µM) for 24 hours. MTT assay was used to assess cell growth. Data are presented as the mean ± SD. **p < 0.01 vs. control; #p < 0.05 vs. PD-treated cells

The effect of PD on apoptosis was investigated using flow cytometry of annexin V/PI-stained cells and western blot analysis of apoptosis-related proteins. The percentage of Annexin V/PI-positive cells increased from 7.0% to 26.0% in GBM8401 cells and from 7.7% to 35.8% in M059K cells following PD treatment (Figure 2A). We observed a significant increase in the protein expression of cleaved-PARP (c-PARP) in PD-treated GBM8401 and M059K cells (Figure 2B). To further confirm these findings, we co-treated glioblastoma cells with PD and the pan-caspase inhibitor Z-VAD. Z-VAD treatment significantly reversed the PD-induced decrease in GBM8401 and M059K cell viability compared to PD treatment alone (Figure-2C). These findings suggest that PD induces glioblastoma cell death via apoptosis.

### PD induced mitochondrial dysfunction in human glioblastoma cells

**Figure 3:**
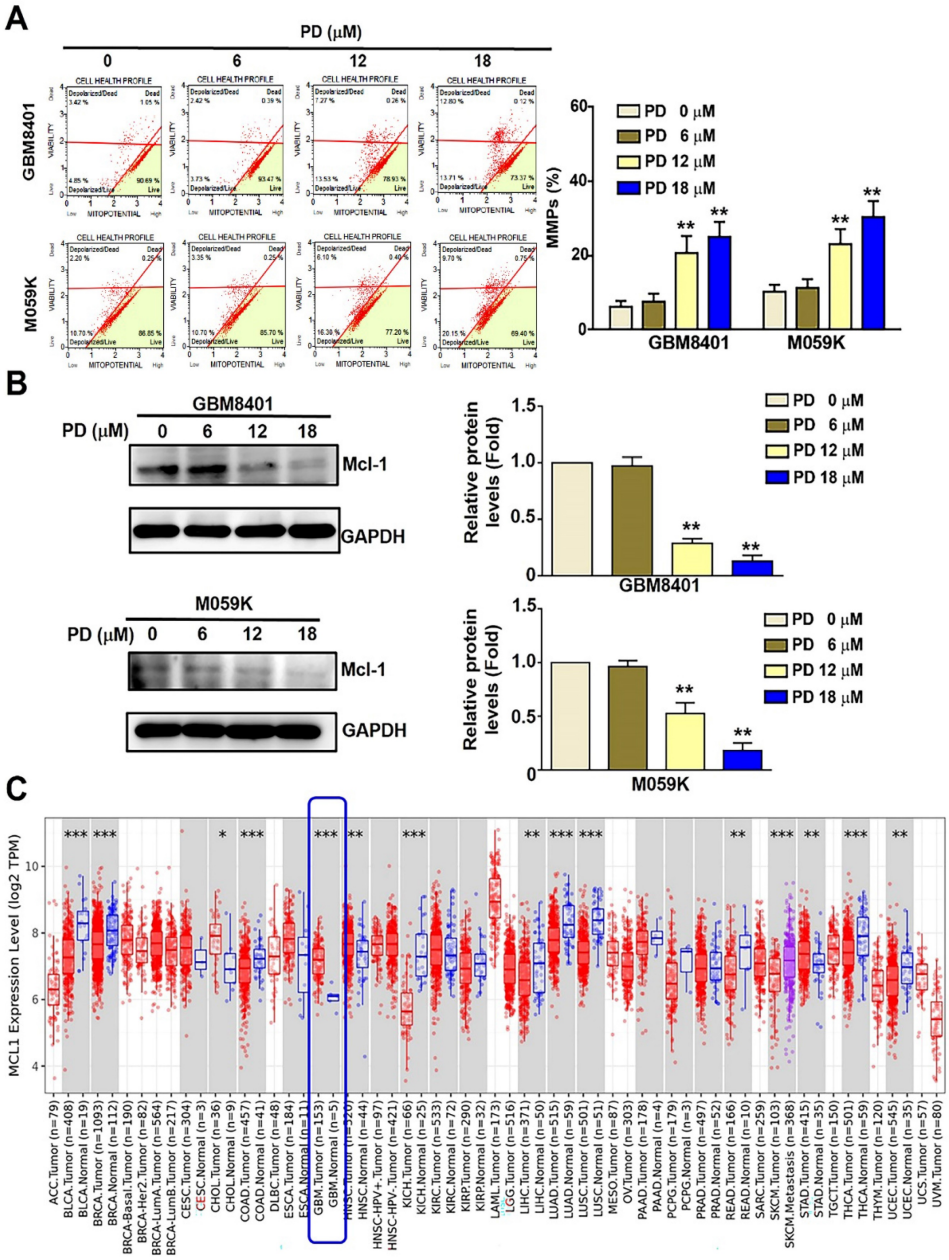
** PD treatment induces mitochondrial dysfunction in human glioblastoma cells.** GBM8401 and M059K cells were treated with various concentrations of PD for 24 hours, and (A) MitoPotential staining was performed to observe to MMPs. Right panel: quantification of MMPs. (B) Western blot analysis of MCL-1 expression. Right panel: quantification of fold-changes in MCL-1/GAPDH. (C) TCGA data analysis using Time2.0 software compared MCL-1 expression in normal and glioblastoma tissues. Data are presented as the mean ± SD. **p < 0.01 vs. control cells.

Mitochondrial membrane dysfunction serves as a critical indicator of apoptosis in mammalian cells 17. In this study, the mitochondrial membrane potential (MMP) of PD-treated glioblastoma cells was assessed using flow cytometry after treatment using the MitoPotential kit. The results showed a significant increase in MMP disruption following PD treatment, rising from 8.27% to 26.51% in GBM8401 cells and from 12.9% to 29.85% in M059K cells (Figure 3A). MCL-1 regulates tumor-cell survival and mitochondrial membrane permeabilization in a variety of tumor cells 18. Western blot analysis revealed that PD significantly reduced MCL-1 protein expression in both glioblastoma cell lines (Figure 3B). Additionally, TCGA database analysis using TIMER 2.0 indicated that MCL-1 expression is significantly higher in glioblastoma tumor tissues (n =153) than in normal tissues (n=5) (Figure 3C). These findings suggest that MCL-1 is a potential target of PD and may be involved in the modulation of apoptosis in human glioblastoma cells.

### PD induces autophagy dependent on mitochondrial apoptosis by targeting LC3 expression in human glioblastoma cells

**Figure 4:**
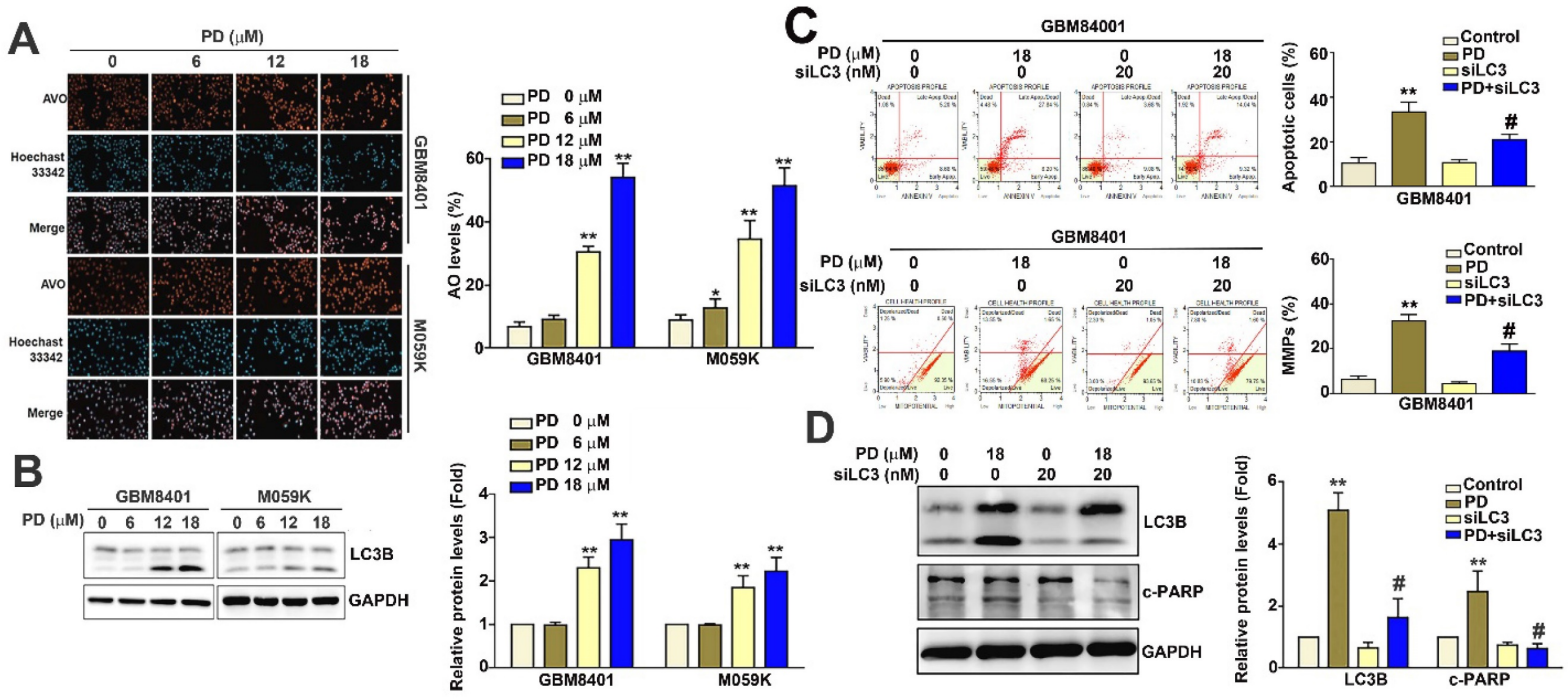
** PD induces autophagy by targeting LC3 in human glioblastoma cells.** (A) GBM8401 and M059K glioblastoma cells were treated with various concentrations of PD for 24 hours, and the formation of acidic vesicular organelles was assessed using acridine orange staining. Total cell numbers were determined by nuclear Hoechst 33342 staining. (B) Western blot analysis of LC3B protein expression in GBM8401 and M059K cells. GAPDH was used as a loading control. Right panel: quantification of fold-changes in LC3B/GAPDH. Cells were pre-treated with siLC3 (20 nM) for 6 hours, then treated with PD (18 μM) for 24 hours. (C) Changes in the number of apoptotic GBM8401 cells and (D) mitochondrial membrane potential was determined by flow cytometric analysis. (E) Western blot analysis of LC3B and cleaved PARP (c-PARP) in GBM8401 cells. Right panel: quantification of fold-changes in c-PARP/GAPDH. Data are presented as the mean ± SD. *p < 0.05; **p < 0.01 vs. control; #p < 0.05 vs. PD-treated cells. Scale bar=50 µm

When cellular autophagy is activated, acridine orange accumulates in acidic vesicles and emits red fluorescence 19. In this study, we used acridine orange staining and fluorescence microscopy to quantitatively assess AVOs and evaluate the effect of PD on autophagolysosome formation in glioblastoma cells. Our results show that PD treatment significantly increased the percentage of AVOs from 7.8% to 57.6% in GBM8401 cells and from 11.2% to 51.5% in M059K cells (Figure 4A). Furthermore, PD markedly upregulated LC3B protein expression in both glioblastoma cell lines (Figure 4B). LC3 knockdown using siRNA (siLC3; 20 nM) led to a reduction in both apoptosis (Figure 4C) and mitochondrial dysfunction (Figure 4D) in PD-treated GBM8401 cells compared to PD treatment alone. LC3 knockdown also decreased the expression of LC3B and cleaved-PARP (c-PARP) in PD-treated GBM8401 cells (Figure 4E). Taken together, these findings suggest that PD induces both autophagy and apoptosis by targeting LC3 expression in human glioblastoma cells.

### PD exhibits antitumor effects *in vivo* in glioblastoma xenograft mice

**Figure 5:**
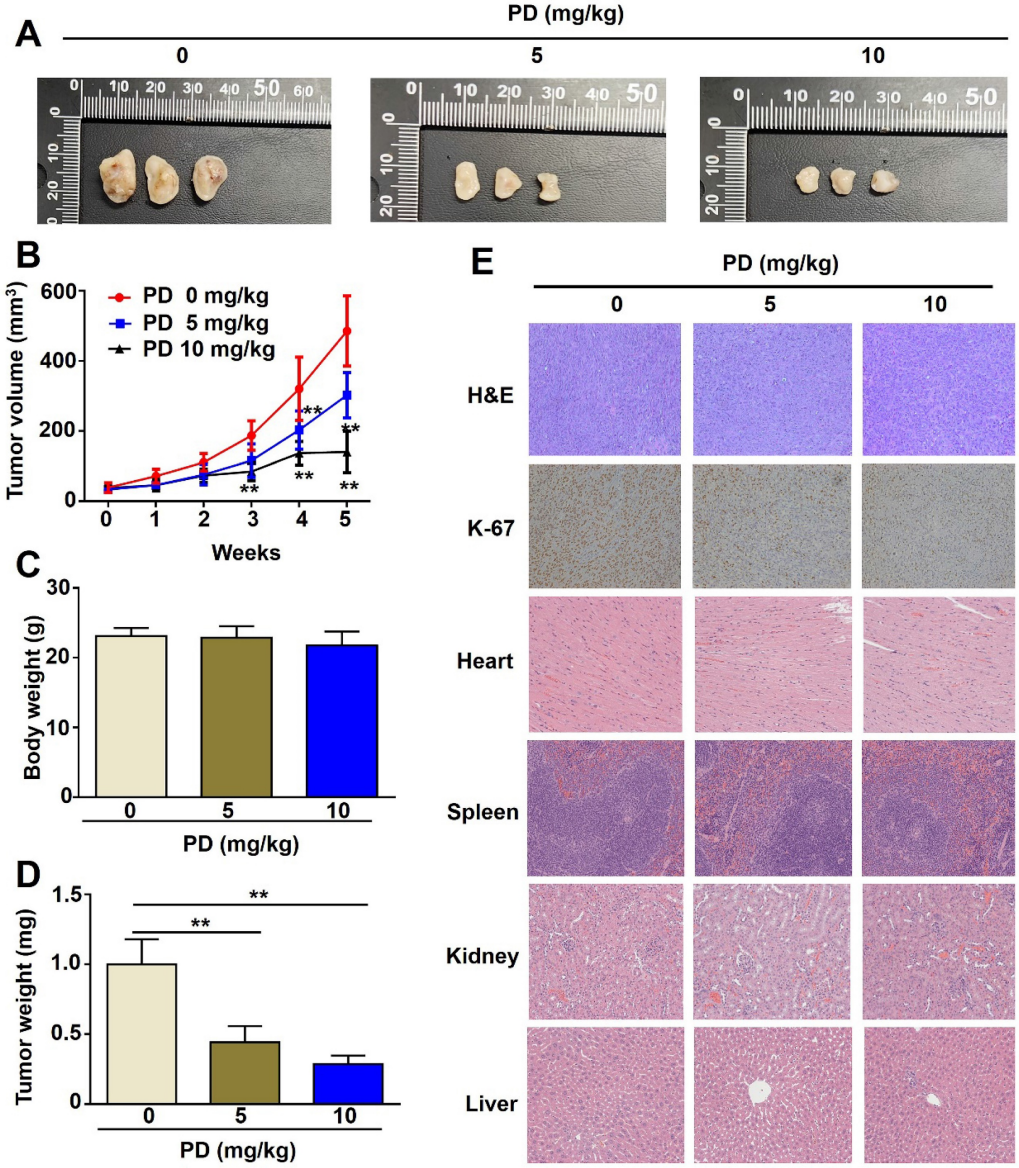
***In vivo* anti-tumor effect of PD in glioblastoma xenograft mice.** (A) GBM8401 cells were subcutaneously injected into five-week-old female BALB/c nude mice, followed by oral administration of different concentrations of PD (5 and 10 mg/kg) for 5 weeks. Representative photographs of tumor-bearing mice are shown (n = 5 per group). (B) Tumor growth curves (tumor volume) under PD treatment were evaluated weekly for 5 weeks. (C) Body weight and (D) tumor weight were measured at the end of the experiment. (E) Tumor morphology was assessed by H&E staining, and Ki-67 expression was evaluated by immunohistochemical analysis. Drug safety was assessed by evaluating PD-induced organ toxicity (heart, spleen, kidney, and liver) using H&E staining. Data are presented as the mean ± SD. *p < 0.05; **p < 0.01 vs. control group. Scale bar=100 µm

GBM8401 cells were subcutaneously implanted into immunodeficient BALB/c nude mice, and tumor-bearing mice were orally administered PD (5 and 10 mg/kg) twice weekly. PD treatment (5 and 10 mg/kg) significantly suppressed tumor growth compared to control mice (Figure-5A, 5B), with a marked reduction in tumor weight (Figure-5D) and no significant difference in body weight between the PD-treated and control mice (Figure-5C). Immunohistochemical analysis of tumors excised at the end of the treatment period showed that PD reduced Ki-67 expression (cell proliferation marker), indicating that PD exerts antitumor effects on glioblastoma cells *in vivo* (Figure 5E). Additionally, histopathological examination of organs from control or PD-treated mice, including the heart, spleen, kidney, and liver, showed no significant organ toxicity (Figure 5E). These results suggest that PD not only reduces glioblastoma tumor growth but also exhibits a favorable safety profile *in vivo*.

### Model predicting molecular docking between PD and LC3B/MCL-1

**Figure 6:**
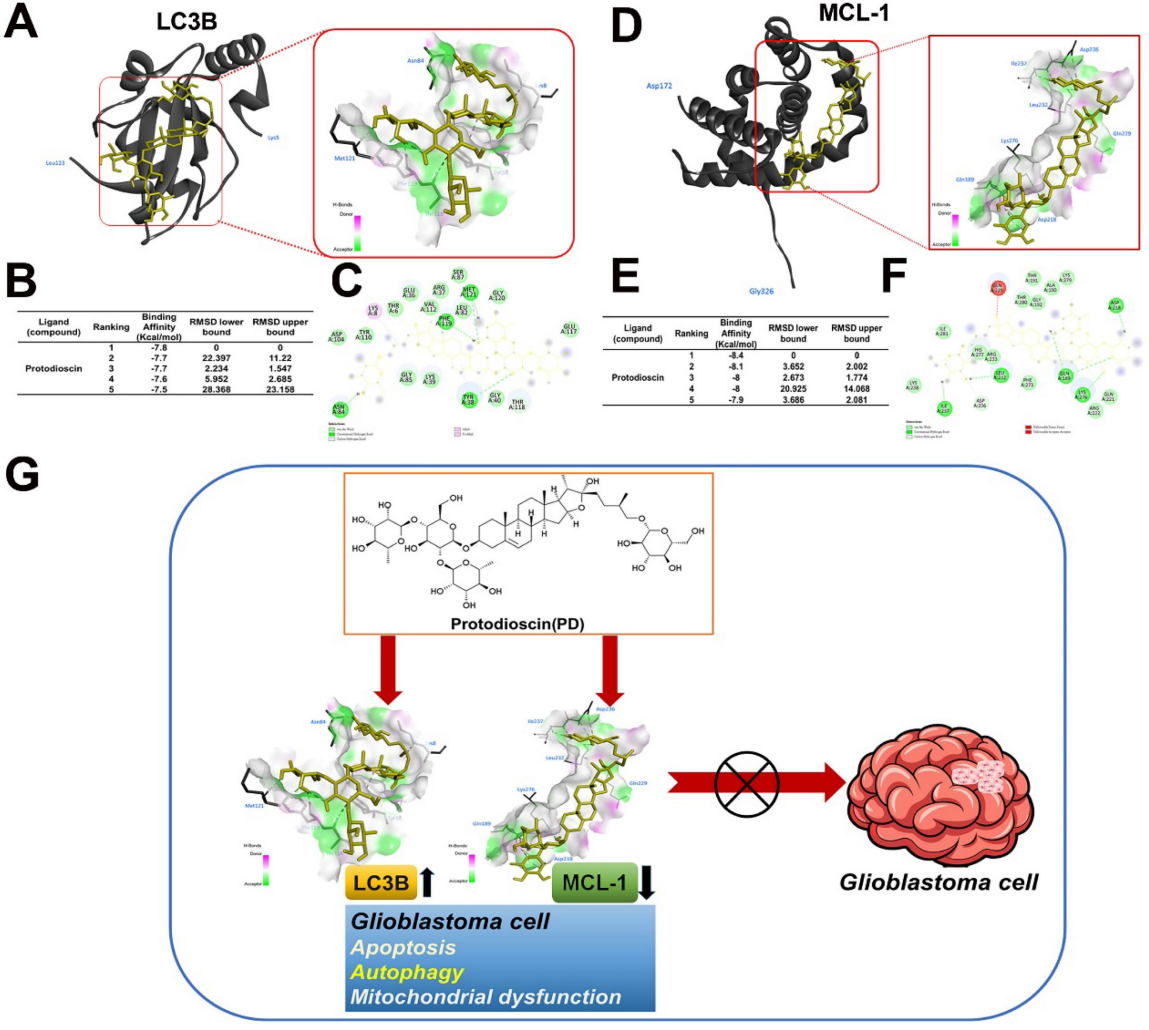
** PD directly targets LC3B/MCL-1 in molecular docking model.** (A, C) The 3D structure of LC3B is shown with PD (yellow) docked into the binding site, indicating strong binding interaction. (D, F) Surface representation of LC3B and MCL-1 with PD bound to a hydrophobic pocket and shown in key interaction residues. (B, E) Docking scores of PD with LC3B and MCL-1 show the highest binding affinity. (G) Schematic diagram illustrates the proposed molecular mechanism of PD targeting LC3B and MCL-1 by which PD exerts anti-glioblastoma effects.

Molecular docking analysis with AutoDock software was used to explore the potential interactions between PD and LC3B/MCL-1. The resulting 3D docking model shows PD binding to the LC3B protein and MCL-1 structure. Figure 6 shows the binding pocket formed in the surface interactions between PD and LC3B (Figure 6A) or MCL-1 (Figure 6D). The docking results show that PD (PDB: 5D94) has a binding affinity of -7.8 kcal/mol for the LC3B protein (Figure 6B) and -8.4 kcal/mol for the MCL-1 protein (Figure 6E). Binding pose analysis of LC3B (PDB: 5D94) shows that the ligand forms stable hydrogen bonds with key residues Tyr38, Asn84, Phe119, and Met121, as well as a C-H bond with Thr118 (Figure 6C); analysis of MCL-1 (PDB: 6UDV) found that the ligand forms stable hydrogen bonds with key residues Gln189, Asp218, Leu232, Ile237 and Lys276, as well as a C-H bond with Asp236 (Figure 6F). These results suggest that PD directly targets LC3B and MCL-1, which may play a critical role in mediating PD-induced autophagy and apoptosis in glioblastoma cells.

## Discussion

Glioblastoma is an aggressive and lethal brain tumor. Despite multimodal treatment strategies combining surgery, radiotherapy, and temozolomide chemotherapy, patient outcomes remain poor, with a median overall survival of approximately 15 months 20. Effective drug treatment remains a major challenge, as resistance to temozolomide develops rapidly. Moreover, other targeted therapies have demonstrated only limited survival benefits in clinical trials. Increasing evidence suggests that natural compounds may serve as an alternative or adjunctive therapeutic strategy against human glioblastoma 21, 22, potentially reducing the toxicity and side effects associated with conventional chemotherapeutic agents and improving treatment outcomes 23, 24. In this study, we propose a novel therapeutic approach, demonstrating that PD exerts potent antitumor effects on glioblastoma cells by targeting LC3B and MCL-1 expression. These findings suggest its potential as a promising agent for glioblastoma treatment (Figure 6G).

PD is reported to exhibit antitumor and pro-apoptotic activities in a variety of tumor cell types. Therefore, evaluation of the effective dosage of PD is essential, as it varies depending on the tumor cell line and is crucial for minimizing toxicity to normal cells. Some studies show that PD induced significant cytotoxic effects at concentrations ranging from 2 to 6 μM in both ER-positive (MCF-7) and ER-negative (MDA-MB-468) breast cancer cells 8. Hu et al. found that PD exhibited potent cytotoxic activity with GI₅₀ values ≤ 2.0 μM in several tumor cell lines, including leukemia cells (MOLT-4), NSCLC cells (A549), colon cancer cells (HCT-116 and SW-620), and renal cancer cells (786-O) 25. Other studies have reported PD-associated cell death effects induced through mitophagy (IC₅₀ values ranging from 2 to 8 μM) in osteosarcoma (OS) cells 7, hepatocellular carcinoma cells 9, and cervical cancer cells 26. Bladder cancer cells (5637 and T24) exhibited higher sensitivity, with significant induction of apoptosis at higher PD dosages of approximately 60-70 μM. Lower doses of PD were sufficient to inhibit bladder cancer cell migration and invasion 27. These findings highlight the importance of optimizing dosage strategies tailored to specific tumor cell types for potential therapeutic applications. In our study, we found that glioblastoma cells (GBM8401, M059K, and U-251) required moderately higher PD dosages, typically 12-18 μM, to achieve comparable levels of cell growth inhibition, apoptosis induction, and autophagy activation. These results further support evidence that the antitumor efficacy of PD is cell type-dependent, underscoring the importance of optimizing its combined effects with chemotherapeutic agents for future potential therapeutic applications and investigations. However, a limitation of this study is the lack of data on primary normal brain cells or human astrocytes. Peripheral blood mononuclear cells (PBMCs) are frequently employed as normal cells to evaluate the cytotoxicity of anticancer agents and are widely used in standard chemotherapy assays. Dr. Bouchmaa et al. reported that PD exhibited low cytotoxicity toward PBMCs, with an IC₅₀ ≥ 80 μM 8. According to the National Cancer Institute (NCI) of the United States, pure compounds are considered cytotoxic when their IC₅₀ values are less than 30 μg/mL. In our study, PD demonstrated potent antitumor effects in three human glioma cell lines, with IC₅₀ values below 24 μM. Further studies are needed to evaluate the cytotoxicity of PD in normal brain cells to assess its therapeutic selectivity.

LC3B plays dual roles in tumor cells. Increased LC3B-mediated autophagy often induces tumor cell death under stress conditions such as hypoxia, nutrient deprivation, and chemotherapy 28, 29. High LC3B expression has been correlated with poor prognosis in pancreatic and breast cancer 30, 31. Zielke et al. found that loperamide, pimozide, and STF-62247 trigger autophagy-dependent cell death in glioblastoma cells, following the induction of autophagy 32. Down-regulation of LC3B expression was observed to be positively associated with glioblastoma progression 33. Studies have shown that PD exerts its anticancer effects through altering LC3B expression in several types of cancer cells. PD induces mitochondrial membrane potential dysfunction and mitophagy by modulating p38MAPK-mediated NIX/LC3 pathways in osteosarcoma cells 7. Additionally, PD has been reported to induce apoptosis and autophagy, contributing to its cytotoxic effects in human hepatocellular carcinoma cells 34.

MCL-1 plays a critical role in the survival of glioblastoma cells by regulating apoptotic pathways. Elevated MCL-1 expression correlates with a poor prognosis in glioblastoma patients. Inhibition of MCL-1 has been shown to increase the sensitivity of glioblastoma cells to chemotherapy and radiotherapy, suggesting that the targeting of MCL-1 could be an effective strategy for treating glioblastoma. Several natural compounds have demonstrated promising effects in modulating MCL-1 to induce cancer cell death. Resveratrol induces mitochondrial apoptosis through the suppression of MCL-1 expression in breast and lung cancer cells 35, 36. Similarly, curcumin promotes both apoptosis and autophagy in hepatocellular carcinoma cells by downregulating MCL-1 expression 37. In the present study, we showed that PD induces apoptosis in glioblastoma cells by downregulating MCL-1 expression and observed a strong predicted interaction between PD and MCL-1 in molecular docking analysis. However, we currently lack direct experimental evidence to confirm the role of MCL-1 in PD-induced mitochondrial apoptotic pathways in glioblastoma cells, which warrants further investigation.

The present study is the first to report that PD induces both apoptosis and autophagy by targeting LC3/MCL-1 in glioblastoma cells both *in vitro* and *in vivo*. Taken together, PD is a promising candidate for glioblastoma therapy and for use in the development of novel therapeutic strategies for cancer treatment.

## References

[1] Siminska D, Korbecki J, Kojder K, Kapczuk P, Fabianska M, Gutowska I (2021). Epidemiology of Anthropometric Factors in Glioblastoma Multiforme-Literature Review. Brain Sci.

[2] Cruz-Martins N (2023). Advances in Plants-Derived Bioactives for Cancer Treatment. Cells.

[3] Tagde P, Tagde P, Tagde S, Bhattacharya T, Garg V, Akter R (2021). Natural bioactive molecules: An alternative approach to the treatment and control of glioblastoma multiforme. Biomed Pharmacother.

[4] Navarro Del Hierro J, Casado-Hidalgo G, Reglero G, Martin D (2021). The hydrolysis of saponin-rich extracts from fenugreek and quinoa improves their pancreatic lipase inhibitory activity and hypocholesterolemic effect. Food Chem.

[5] He X, Qiao A, Wang X, Liu B, Jiang M, Su L (2006). Structural identification of methyl protodioscin metabolites in rats' urine and their antiproliferative activities against human tumor cell lines. Steroids.

[6] Passos FRS, Araujo-Filho HG, Monteiro BS, Shanmugam S, Araujo AAS, Almeida J (2022). Anti-inflammatory and modulatory effects of steroidal saponins and sapogenins on cytokines: A review of pre-clinical research. Phytomedicine.

[7] Huang CF, Hsieh YH, Yang SF, Kuo CH, Wang PH, Liu CJ (2023). Mitophagy Effects of Protodioscin on Human Osteosarcoma Cells by Inhibition of p38MAPK Targeting NIX/LC3 Axis. Cells.

[8] Bouchmaa N, Ben Mrid R, Bouargalne Y, Ajouaoi S, Cacciola F, El Fatimy R (2023). *In vitro* evaluation of dioscin and protodioscin against ER-positive and triple-negative breast cancer. PLoS One.

[9] Yu CL, Lee HL, Yang SF, Wang SW, Lin CP, Hsieh YH (2022). Protodioscin Induces Mitochondrial Apoptosis of Human Hepatocellular Carcinoma Cells Through Eliciting ER Stress-Mediated IP3R Targeting Mfn1/Bak Expression. J Hepatocell Carcinoma.

[10] Jacquet M, Guittaut M, Fraichard A, Despouy G (2021). The functions of Atg8-family proteins in autophagy and cancer: linked or unrelated?. Autophagy.

[11] Liu S, Yao S, Yang H, Liu S, Wang Y (2023). Autophagy: Regulator of cell death. Cell Death Dis.

[12] Chen Y, Yi H, Liao S, He J, Zhou Y, Lei Y (2025). LC3B: A microtubule-associated protein influences disease progression and prognosis. Cytokine Growth Factor Rev.

[13] Dikic I, Elazar Z (2018). Mechanism and medical implications of mammalian autophagy. Nat Rev Mol Cell Biol.

[14] Chiang YF, Huang KC, Chen HY, Hamdy NM, Huang TC, Chang HY (2024). Hinokitiol Inhibits Breast Cancer Cells *In Vitro* Stemness-Progression and Self-Renewal with Apoptosis and Autophagy Modulation via the CD44/Nanog/SOX2/Oct4 Pathway. Int J Mol Sci.

[15] Chen Y-J, Chien P-H, Chen W-S, Chien Y-F, Hsu Y-Y, Wang L-Y (2013). Hepatitis B Virus-Encoded X Protein Downregulates EGFR Expression via Inducing MicroRNA-7 in Hepatocellular Carcinoma Cells. Evid Based Complement Alternat Med.

[16] Sun WC, Lin CL, Lee TH, Chang CH, Ong AZ, Yeh YH (2023). Critical role of heme oxygenase-1 in chaetoglobosin A by triggering reactive oxygen species mediated mitochondrial apoptosis in colorectal cancer. Free Radic Biol Med.

[17] Li RL, Wang LY, Duan HX, Zhang Q, Guo X, Wu C (2022). Regulation of mitochondrial dysfunction induced cell apoptosis is a potential therapeutic strategy for herbal medicine to treat neurodegenerative diseases. Front Pharmacol.

[18] Wang H, Guo M, Wei H, Chen Y (2021). Targeting MCL-1 in cancer: current status and perspectives. J Hematol Oncol.

[19] Thome MP, Filippi-Chiela EC, Villodre ES, Migliavaca CB, Onzi GR, Felipe KB (2016). Ratiometric analysis of Acridine Orange staining in the study of acidic organelles and autophagy. J Cell Sci.

[20] Rana M, Liou KC, Thakur A, Nepali K, Liou JP (2025). Advancing glioblastoma therapy: Learning from the past and innovations for the future. Cancer Lett.

[21] Luo C, Chen G, Li R, Peng S, Zhang P, Wang F (2024). Juglone suppresses vasculogenic mimicry in glioma through inhibition of HuR-mediated VEGF-A expression. Biochem Pharmacol.

[22] Ling Z, Pan J, Zhang Z, Chen G, Geng J, Lin Q (2024). Small-molecule Molephantin induces apoptosis and mitophagy flux blockage through ROS production in glioblastoma. Cancer Lett.

[23] Beltzig L, Christmann M, Kaina B (2022). Abrogation of Cellular Senescence Induced by Temozolomide in Glioblastoma Cells: Search for Senolytics. Cells.

[24] Cui P, Chen F, Ma G, Liu W, Chen L, Wang S (2022). Oxyphyllanene B overcomes temozolomide resistance in glioblastoma: Structure-activity relationship and mitochondria-associated ER membrane dysfunction. Phytomedicine.

[25] Hu K, Yao X (2002). Protodioscin (NSC-698 796): its spectrum of cytotoxicity against sixty human cancer cell lines in an anticancer drug screen panel. Planta Med.

[26] Lin CL, Lee CH, Chen CM, Cheng CW, Chen PN, Ying TH (2018). Protodioscin Induces Apoptosis Through ROS-Mediated Endoplasmic Reticulum Stress via the JNK/p38 Activation Pathways in Human Cervical Cancer Cells. Cell Physiol Biochem.

[27] Chen YR, Wang SC, Huang SP, Su CC, Liu PL, Cheng WC (2022). Protodioscin inhibits bladder cancer cell migration and growth, and promotes apoptosis through activating JNK and p38 signaling pathways. Biomed Pharmacother.

[28] Sun C, Gao X, Sha S, Wang S, Shan Y, Li L (2025). Berberine alleviates Alzheimer's disease by activating autophagy and inhibiting ferroptosis through the JNK-p38MAPK signaling pathway. Int Immunopharmacol.

[29] Xing Y, Lv X, Chen X, Du J, Hu D, He R (2025). Maackiain induces apoptosis and autophagy via ROS-mediated endoplasmic reticulum stress in endometrial cancer. Int Immunopharmacol.

[30] Odermatt M, Miskovic D, Siddiqi N, Khan J, Parvaiz A (2013). Short- and long-term outcomes after laparoscopic versus open emergency resection for colon cancer: an observational propensity score-matched study. World J Surg.

[31] Bortnik S, Tessier-Cloutier B, Leung S, Xu J, Asleh K, Burugu S (2020). Differential expression and prognostic relevance of autophagy-related markers ATG4B, GABARAP, and LC3B in breast cancer. Breast Cancer Res Treat.

[32] Zielke S, Meyer N, Mari M, Abou-El-Ardat K, Reggiori F, van Wijk SJL (2018). Loperamide, pimozide, and STF-62247 trigger autophagy-dependent cell death in glioblastoma cells. Cell Death Dis.

[33] Huang X, Bai HM, Chen L, Li B, Lu YC (2010). Reduced expression of LC3B-II and Beclin 1 in glioblastoma multiforme indicates a down-regulated autophagic capacity that relates to the progression of astrocytic tumors. J Clin Neurosci.

[34] Okubo S, Ohta T, Shoyama Y, Uto T (2021). Steroidal Saponins Isolated from the Rhizome of Dioscorea tokoro Inhibit Cell Growth and Autophagy in Hepatocellular Carcinoma Cells. Life (Basel).

[35] Zhang W, Wang X, Chen T (2012). Resveratrol induces apoptosis via a Bak-mediated intrinsic pathway in human lung adenocarcinoma cells. Cell Signal.

[36] Kotha A, Sekharam M, Cilenti L, Siddiquee K, Khaled A, Zervos AS (2006). Resveratrol inhibits Src and Stat3 signaling and induces the apoptosis of malignant cells containing activated Stat3 protein. Mol Cancer Ther.

[37] Chen WT, Hsu FT, Liu YC, Chen CH, Hsu LC, Lin SS (2019). Fluoxetine Induces Apoptosis through Extrinsic/Intrinsic Pathways and Inhibits ERK/NF-kappaB-Modulated Anti-Apoptotic and Invasive Potential in Hepatocellular Carcinoma Cells *In Vitro*. Int J Mol Sci.

